# Supplementary figures and images for: Effect of environmental DNA sampling resolution in detecting nearshore fish biodiversity compared to capture surveys
Source: PeerJ. 2024 Oct 14;12:e17967. doi: 10.7717/peerj.17967 (PMC11485132; doi:10.7717/peerj.17967)

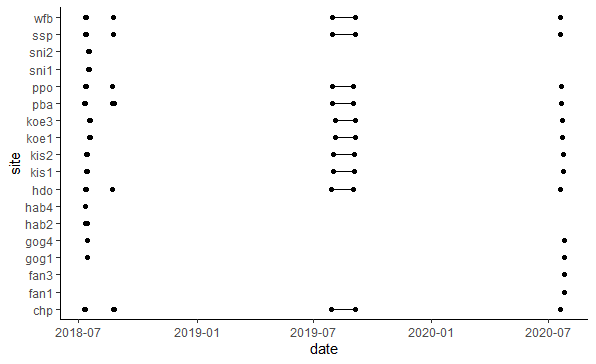

Supplement: Supplemental Information 1 — Pairs of points connected by a solid line indicate paired eDNA and beach seine surveys. Due to closeness in time of most sampling events (2018 and 2020), only a single point or closely overlapping points are visible. [file peerj-12-17967-s001.png]

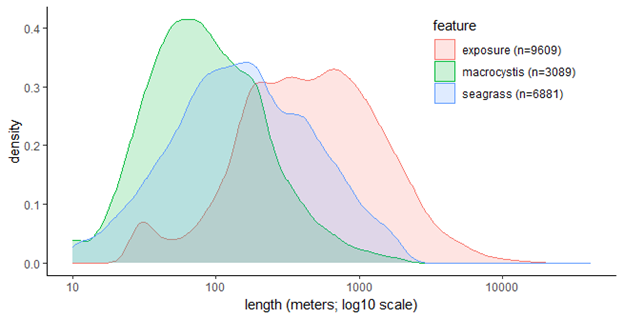

Supplement: Supplemental Information 2 — Exposure feature lengths are the lengths of shoreline not varying in exposure class (mean = 462 m, 10th and 90th percentiles = 112 and 1894 m). Macrocystis and seagrass lengths were derived from maps of Macrocystis pyrifera (mean = 80 m, 10th and 90th percentiles = 27 and 299 m). and Zostera marina (mean = 137 m, 10th and 90th percentiles = 15 and 746 m). distribution. Lengths were the greatest cross-sectional dimension of each discrete vegetation patch calculated in QGIS. [file peerj-12-17967-s002.png]

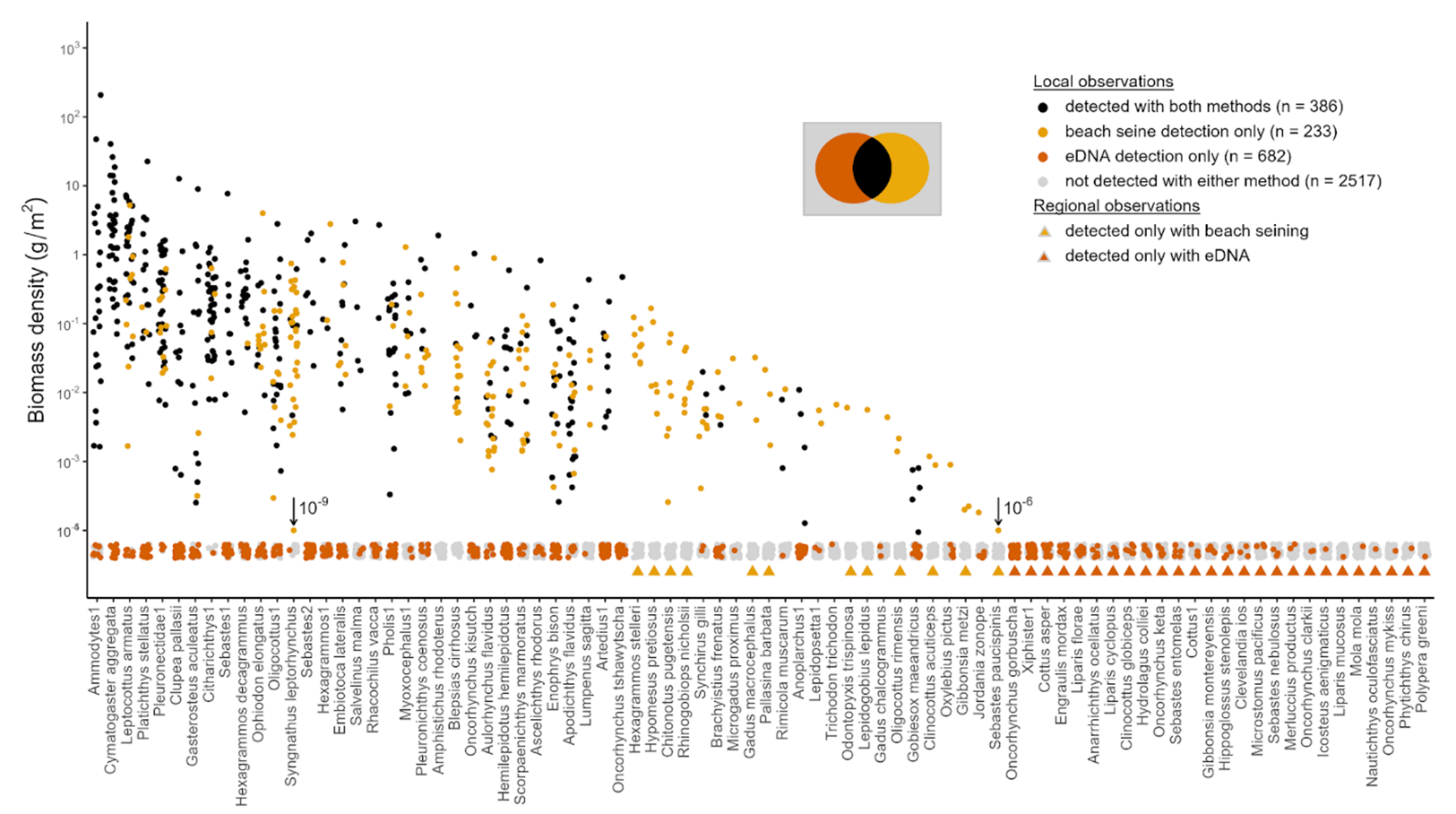

Supplement: Supplemental Information 3 — Biomass density (grams / m2) estimates of all taxa for each paired survey. Biomass density is a summed across two beach seine set replicates. Black points indicate bouts where the taxon was detected in both methods. Yellow and orange points indicate bouts where a taxon was only detected in beach seine or eDNA surveys, respectively. Grey points indicate bouts where the taxon was not detected with either beach seine or eDNA. Yellow and orange triangles indicate taxon detected only in beach seine surveys and eDNA surveys, respectively. [file peerj-12-17967-s003.png]

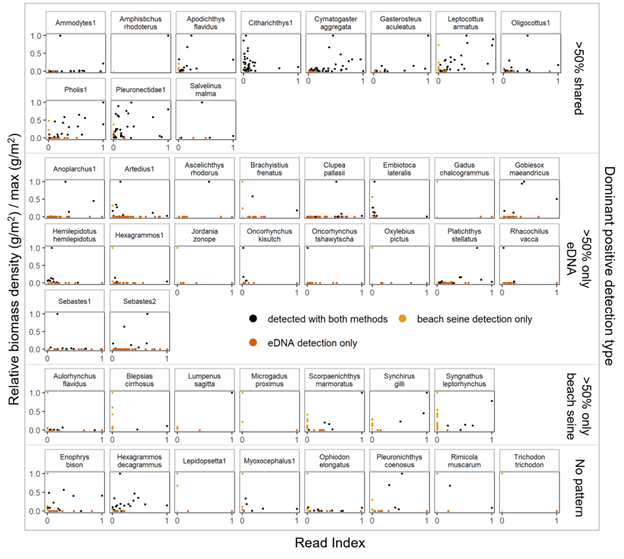

Supplement: Supplemental Information 4 — Each point represents an observation of an taxa in a given paired eDNA/ beach seine survey. Relative biomass density is biomass density scaled to the greatest value for a taxon. Read index is a survey read abundance relative to the total read abundance across that taxa. Black points indicate observations where the taxa was detected in both methods. Orange and red points indicate observations where a taxon was only detected in beach seine or eDNA surveys, respectively. Grey points indicate bouts where the taxon was not detected with either beach seine or eDNA. We grouped taxa based on the dominant positive detection type: taxa that are mostly (> 50%) detected with both methods, taxa that are mostly detected with beach seine, taxa that are mostly detected with eDNA, and taxa that are not primarily detected with both methods, or with either eDNA or beach seining. [file peerj-12-17967-s004.png]

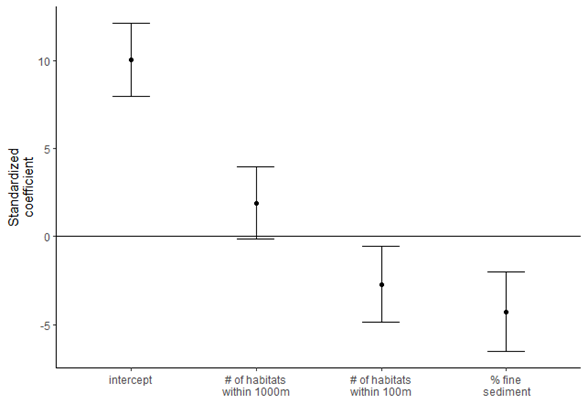

Supplement: Supplemental Information 5 — Richness difference was calculated as the eDNA taxonomic richness minus beach seine taxonomic richness. [file peerj-12-17967-s005.png]

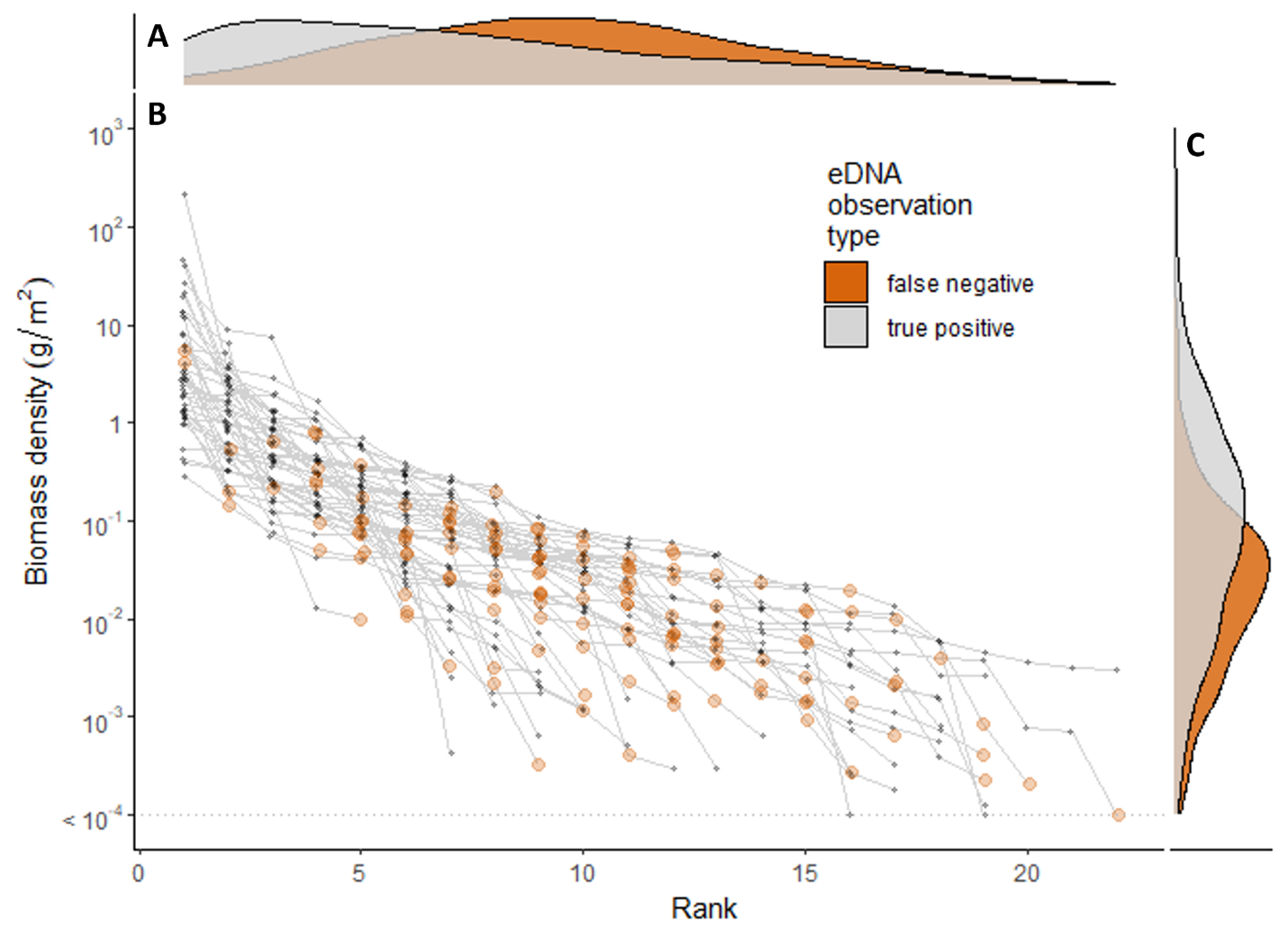

Supplement: Supplemental Information 6 — (A) Distribution of site-level rank biomass density of taxa from beach seines detected by eDNA (true positives) vs. not detected in eDNA (false negatives). (B) Rank biomass densities of each beach seine survey (connected by lines). Colour indicates taxa that were also observed with eDNA (eDNA true positives) vs. not observed with eDNA (eDNA false negative). (C) Distribution of biomass density among eDNA true positives and eDNA false positives. [file peerj-12-17967-s006.png]

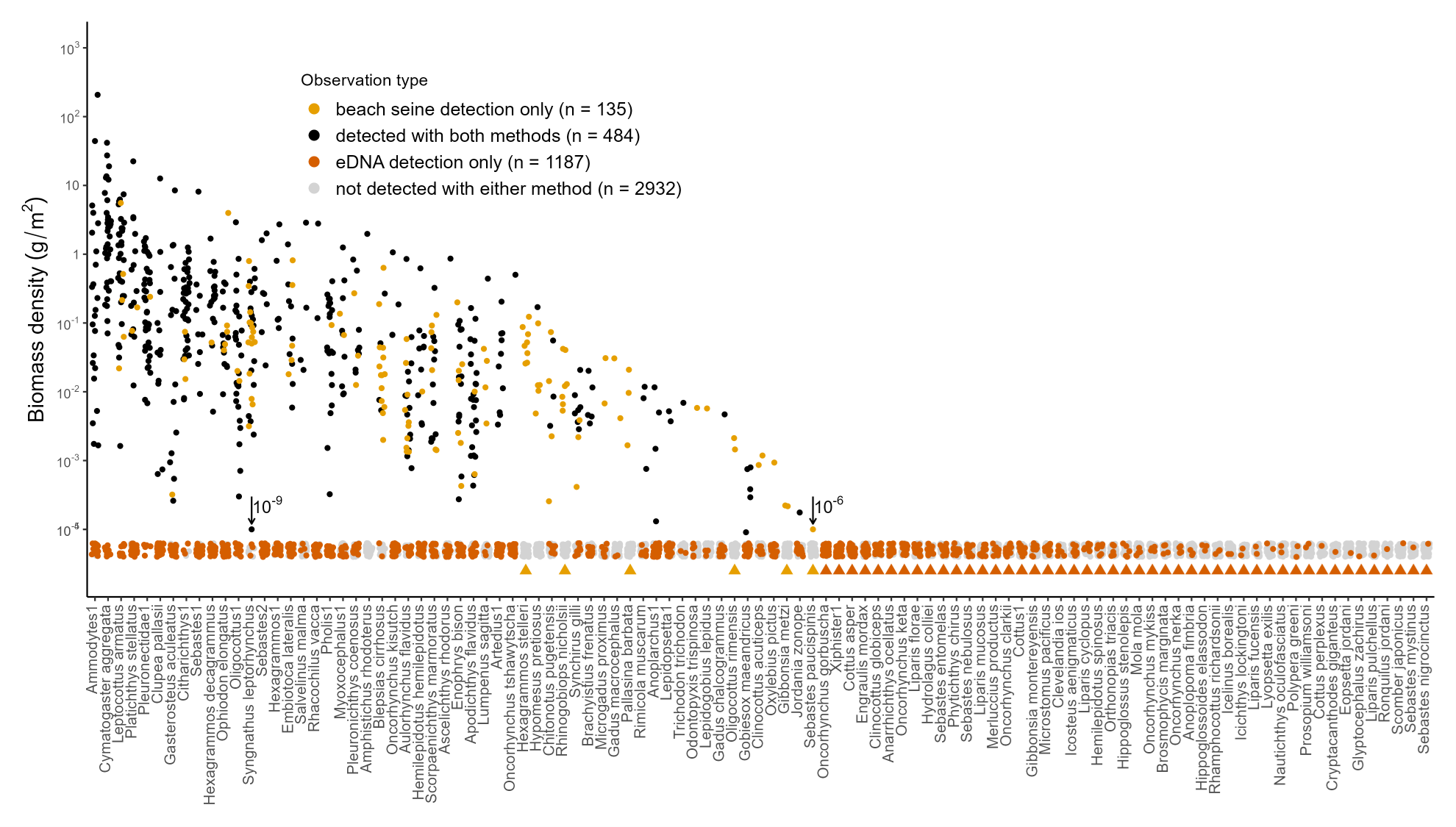

Supplement: Supplemental Information 7 — Biomass density (grams/m2) estimates of all taxa for each paired survey. Biomass density is a summed across two beach seine set replicates. Black points indicate bouts where the taxon was detected in both methods. Yellow and orange points indicate bouts where a taxon was only detected in beach seine or eDNA surveys, respectively. Grey points indicate bouts where the taxon was not detected with either beach seine or eDNA. Yellow and orange triangles indicate taxon detected only in beach seine surveys and eDNA surveys, respectively. [file peerj-12-17967-s007.png]

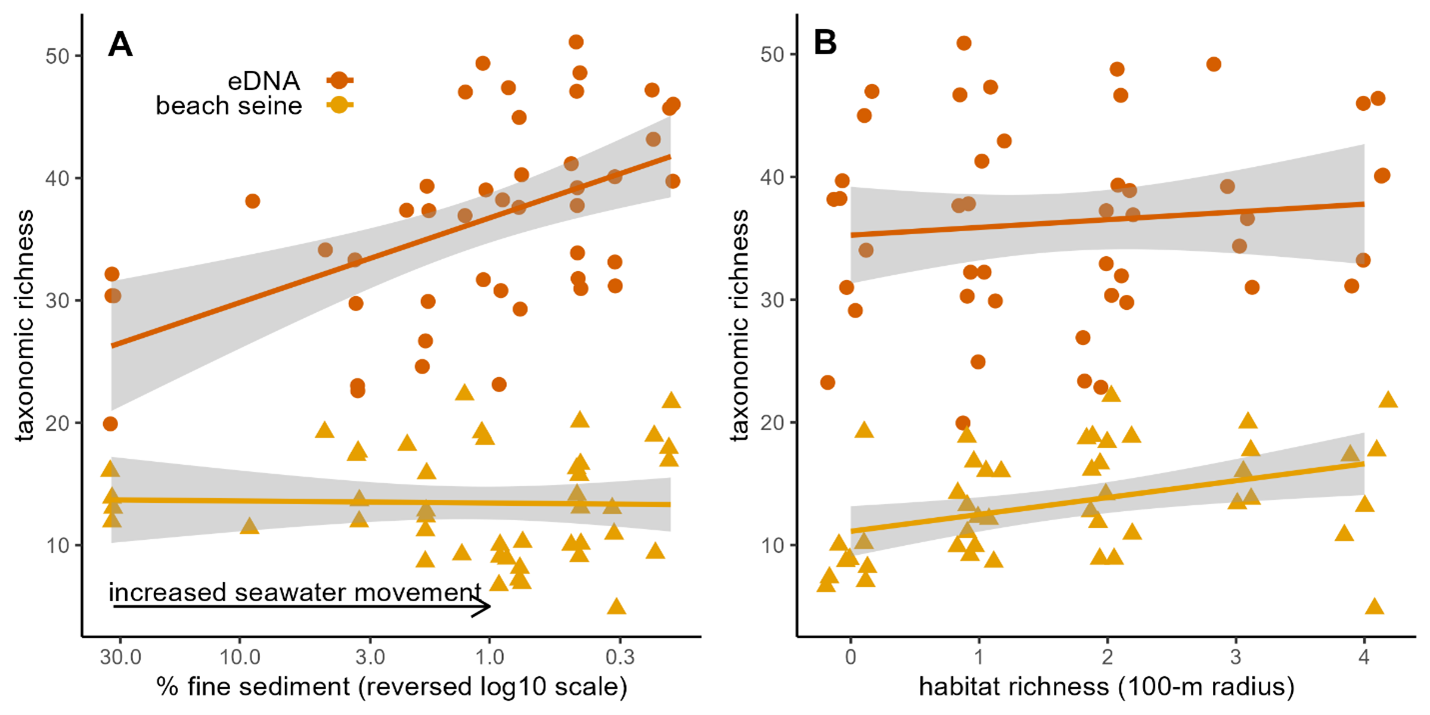

Supplement: Supplemental Information 8 — Circles indicate eDNA richness, triangles indicate beach seining richness. Lines represent post-hoc linear regression models of eDNA and beach seine richness and shaded areas show standard error. Silt percentage is used here as an inverse indicator of water movement. [file peerj-12-17967-s008.png]

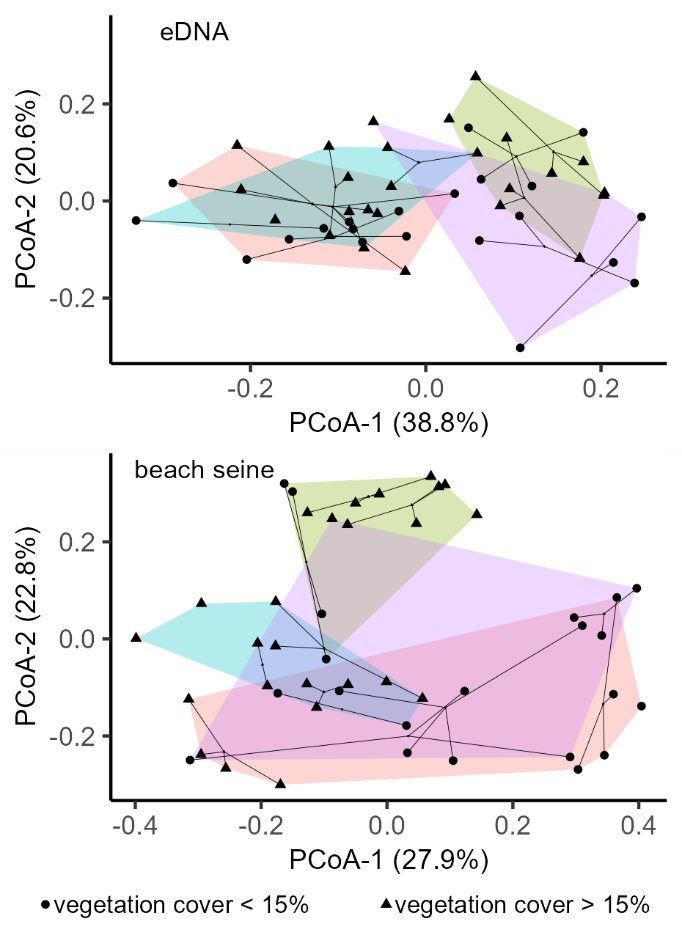

Supplement: Supplemental Information 9 — Colored ellipses in both tiles refer to sub-regions within the study area and correspond to those in Figure 2: Northern-exposed (blue), Northern-protected (purple), Southern-exposed (orange), and Southern-protected (green). [file peerj-12-17967-s009.png]

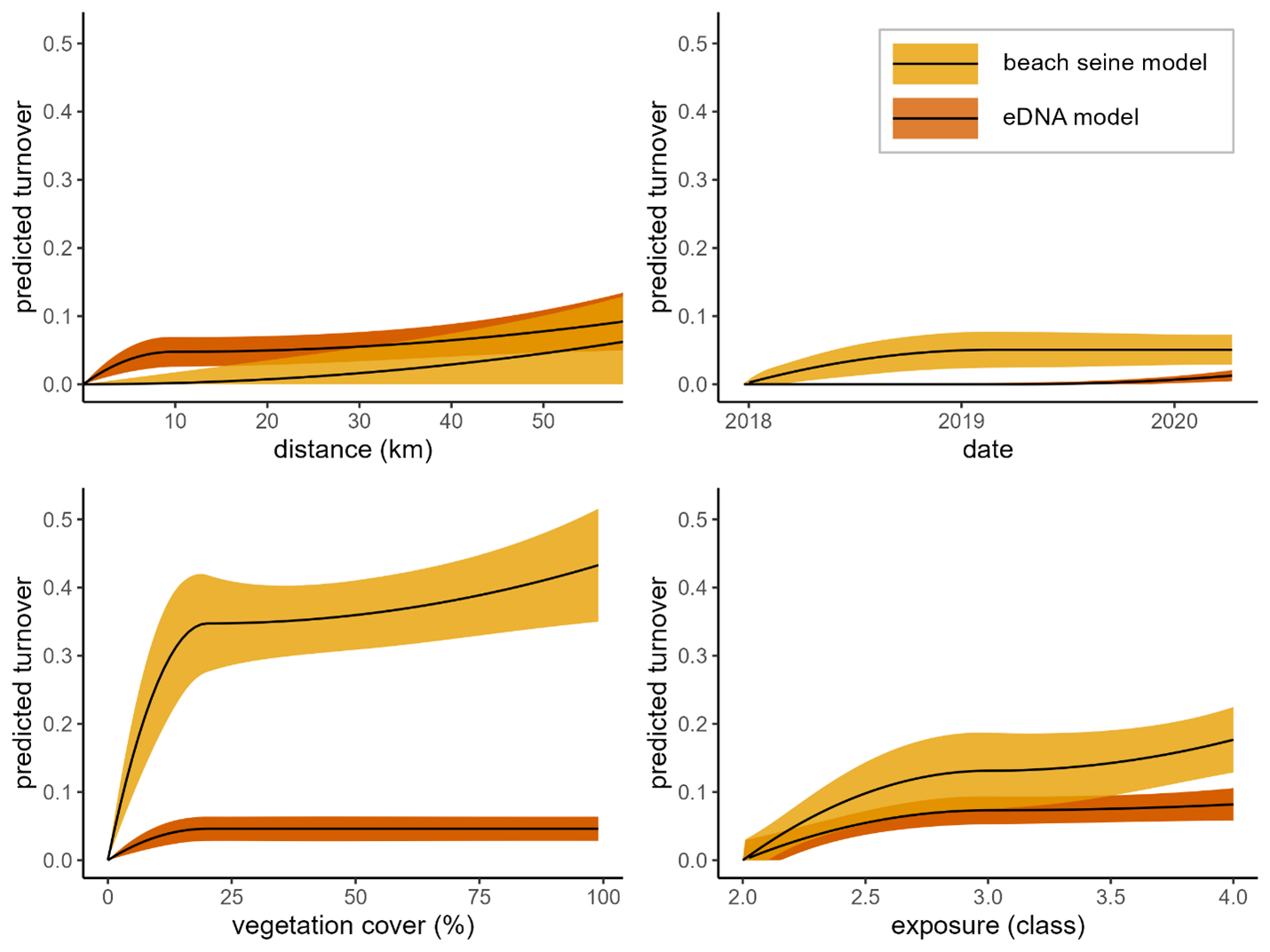

Supplement: Supplemental Information 10 — Lines with error bands (+/- 1 standard deviation) represent i-splines of partial effects of factors across their gradients. Variables were (pairwise distance-over-water), physical ocean exposure class, vegetation percent cover, and Julian data beginning on January 1, 2018. Except for spatial distance, the slope between any two locations along the line indicates the estimated rate of turnover across their range, e.g., beach seining turnover in response to vegetation cover is estimated at around 0.35 (or 35%) between sites with 0% and 25% cover, and near 0 between sites with 25% and 50%. With spatial distance however, rates of turnover are always assessed starting at the origin –one site at the origin and another at a certain distance along the x-axis. [file peerj-12-17967-s010.png]
