# Supplementary material for: Effect of environmental DNA sampling resolution in detecting nearshore fish biodiversity compared to capture surveys
Source: PeerJ. 2024 Oct 14;12:e17967. doi: 10.7717/peerj.17967 (PMC11485132; doi:10.7717/peerj.17967)
Supplement: Supplemental Information 11 [file peerj-12-17967-s011.docx]

| site | eDNA date | beach seine date | latitude | longitude |
| --- | --- | --- | --- | --- |
| chp | 2018-07-12 | 2018-07-10 | 51.67636 | 128.12405 |
| chp | 2018-08-24 | 2018-08-25 | 51.67636 | 128.12405 |
| chp | 2019-09-05 | 2019-07-29 | 51.67636 | 128.12405 |
| chp | 2020-07-19 | 2020-07-19 | 51.67636 | 128.12405 |
| fan1 | 2020-07-25 | 2020-07-25 | 52.0607 | 128.41402 |
| fan3 | 2020-07-25 | 2020-07-25 | 52.05347 | 128.40262 |
| gog1 | 2018-07-15 | 2018-07-14 | 51.92505 | 128.46851 |
| gog1 | 2020-07-26 | 2020-07-26 | 51.92505 | 128.46851 |
| gog4 | 2018-07-15 | 2018-07-14 | 51.90397 | 128.43958 |
| gog4 | 2020-07-26 | 2020-07-26 | 51.90397 | 128.43958 |
| hab2 | 2018-07-15 | 2018-07-12 | 51.66382 | 128.1368 |
| hab4 | 2018-07-12 | 2018-07-11 | 51.665297 | 128.07896 |
| hdo | 2018-07-13 | 2018-07-11 | 51.65456 | 128.13007 |
| hdo | 2018-08-23 | 2018-08-23 | 51.65456 | 128.13007 |
| hdo | 2019-09-02 | 2019-07-29 | 51.65456 | 128.13007 |
| hdo | 2020-07-19 | 2020-07-19 | 51.65456 | 128.13007 |
| kis1 | 2018-07-15 | 2018-07-13 | 51.810975 | 128.22937 |
| kis1 | 2019-09-03 | 2019-08-01 | 51.810975 | 128.22937 |
| kis1 | 2020-07-24 | 2020-07-24 | 51.810975 | 128.22937 |
| kis2 | 2018-07-15 | 2018-07-13 | 51.83706 | 128.23918 |
| kis2 | 2019-09-03 | 2019-08-01 | 51.83706 | 128.23918 |
| kis2 | 2020-07-24 | 2020-07-24 | 51.83706 | 128.23918 |
| koe1 | 2018-07-20 | 2018-07-18 | 51.77404 | 127.87433 |
| koe1 | 2019-09-04 | 2019-08-04 | 51.77404 | 127.87433 |
| koe1 | 2020-07-23 | 2020-07-23 | 51.77404 | 127.87433 |
| koe3 | 2018-07-20 | 2018-07-18 | 51.7756 | 127.87876 |
| koe3 | 2019-09-04 | 2019-08-04 | 51.7756 | 127.87876 |
| koe3 | 2020-07-23 | 2020-07-23 | 51.7756 | 127.87876 |
| pba | 2018-07-12 | 2018-07-10 | 51.64581 | 128.11928 |
| pba | 2018-08-23 | 2018-08-25 | 51.64581 | 128.11928 |
| pba | 2019-09-02 | 2019-07-31 | 51.64581 | 128.11928 |
| pba | 2020-07-21 | 2020-07-21 | 51.64581 | 128.11928 |
| ppo | 2018-07-13 | 2018-07-11 | 51.65768 | 128.11253 |
| ppo | 2018-08-23 | 2018-08-23 | 51.65768 | 128.11253 |
| ppo | 2019-09-02 | 2019-07-31 | 51.65768 | 128.11253 |
| ppo | 2020-07-21 | 2020-07-21 | 51.65768 | 128.11253 |
| sni1 | 2018-07-18 | 2018-07-16 | 51.83842 | 128.05959 |
| sni2 | 2018-07-18 | 2018-07-16 | 51.81764 | 128.03004 |
| ssp | 2018-07-13 | 2018-07-12 | 51.680508 | 128.11521 |
| ssp | 2018-08-24 | 2018-08-24 | 51.680508 | 128.11521 |
| ssp | 2019-09-05 | 2019-07-30 | 51.680508 | 128.11521 |
| ssp | 2020-07-20 | 2020-07-20 | 51.680508 | 128.11521 |
| wfb | 2018-07-13 | 2018-07-12 | 51.66825 | 128.11855 |
| wfb | 2018-08-24 | 2018-08-24 | 51.66825 | 128.11855 |
| wfb | 2019-09-05 | 2019-07-30 | 51.66825 | 128.11855 |
| wfb | 2020-07-20 | 2020-07-20 | 51.66825 | 128.11855 |
