# Supplementary material for: Effect of environmental DNA sampling resolution in detecting nearshore fish biodiversity compared to capture surveys
Source: PeerJ. 2024 Oct 14;12:e17967. doi: 10.7717/peerj.17967 (PMC11485132; doi:10.7717/peerj.17967)
Supplement: Supplemental Information 12 — The source of the partial identification and the method(s) that detected each lowest identifiable taxa are provided as well as the expectation (y/n) of the lowest identifiable taxa’s use of sampled habitat (nearshore soft sediment). [file peerj-12-17967-s012.docx]

| **Lowest Identifiable Taxa name** | **Common name(s)** | **Species name(s)** | **Source of partial ID** | **Method(s) of detection** | **Expected in sampled habitat** |
| --- | --- | --- | --- | --- | --- |
| Ammodytes1 | Pacific sandlance | Ammodytes hexapterus, Ammodytes personatus | eDNA | both | y |
| Cymatogaster aggregata | shiner surfperch | Cymatogaster aggregata |  | both | y |
| Gasterosteus aculeatus | Three-spined stickleback | Gasterosteus aculeatus |  | both | y |
| Leptocottus armatus | Staghorn sculpin | Leptocottus armatus |  | both | y |
| Oligocottus1 | Tidepool and fluffy sculpin | Oligocottus maculosus, Oligocottus snyderi | both | both | y |
| Salvelinus malma | Dolly Varden | Salvelinus malma |  | both | n |
| Amphistichus rhodoterus | Red-tailed surfperch | Amphistichus rhodoterus |  | both | y |
| Pleuronectidae1 | Butter, english, and sand sole | Isopsetta isolepis, Parophrys vetulus, Psettichthys melanostictus | eDNA | both | y |
| Citharichthys1 | Pacific and speckled sanddab | Citharichthys sordidus, Citharichthys stigmaeus | seine | both | y |
| Pholis1 | Longfin and crescent gunnel | Pholis clemensi, Pholis laeta | eDNA | both | y |
| Apodichthys flavidus | penpoint gunnel | Apodichthys flavidus |  | both | y |
| Aulorhynchus flavidus | Tubesnout | Aulorhynchus flavidus |  | both | y |
| Syngnathus leptorhynchus | Bay pipefish | Syngnathus leptorhynchus |  | both | y |
| Lumpenus sagitta | Snake prickleback | Lumpenus sagitta |  | both | y |
| Blepsias cirrhosus | Sinverspotted sculpin | Blepsias cirrhosus |  | both | y |
| Scorpaenichthys marmoratus | Cabezon | Scorpaenichthys marmoratus |  | both | y |
| Microgadus proximus | Pacific tomcod | Microgadus proximus |  | both | y |
| Synchirus gilli | Manacled sculpin | Synchirus gilli |  | both | y |
| Platichthys stellatus | Starry flounder | Platichthys stellatus |  | both | y |
| Clupea pallasii | Pacific Herring | Clupea pallasii |  | both | y |
| Sebastes1 | Copper and quillback rockfish | Sebastes caurinus, Sebastes maliger | eDNA | both | y |
| Rhacochilus vacca | Pile Perch | Rhacochilus vacca |  | both | y |
| Hexagrammos1 | Rock and masked greenling | Hexagrammos lagocephalus, Hexagrammos octogrammus | eDNA | both | y |
| Embiotoca lateralis | striped surfperch | Embiotoca lateralis |  | both | y |
| Sebastes2 | see discussion | Sebastes - alutianus, alutus, elongatus, flavidus, melanistictus, melanops, ruberrimus |  | both | na |
| Oncorhynchus kisutch | Coho salmon | Oncorhynchus kisutch |  | both | n |
| Ascelichthys rhodorus | Rosylip sculpin | Ascelichthys rhodorus |  | both | n |
| Hemilepidotus hemilepidotus | Red Irish lord | Hemilepidotus hemilepidotus |  | both | n |
| Oncorhynchus tshawytscha | Chinook salmon | Oncorhynchus tshawytscha |  | both | n |
| Artedius1 | Padded, scaleyhead, and smooth sculpin | Artedius fenestralis, Artedius harringtoni, Artedius lateralis | seine | both | y |
| Brachyistius frenatus | Kelp perch | Brachyistius frenatus |  | both | y |
| Anoplarchus1 | Slender and high coxcomb | Anoplarchus insignis, Anoplarchus purpurescens | eDNA | both | y |
| Gadus chalcogrammus | Walleyed pollock | Gadus chalcogrammus |  | both | y |
| Oxylebius pictus | Painted greenling | Oxylebius pictus |  | both | y |
| Gobiesox maeandricus | Northern clingfish | Gobiesox maeandricus |  | both | y |
| Jordania zonope | Longfin sculpin | Jordania zonope |  | both | n |
| Ophiodon elongatus | Lingcod | Ophiodon elongatus |  | both | y |
| Hexagrammos decagrammus | Kelp greenling | Hexagrammos decagrammus |  | both | y |
| Myoxocephalus1 | Great and Stellar's sculpin | Myoxocephalus polyacanthocephalus, Myoxocephalus stelleri | eDNA | both | y |
| Pleuronichthys coenosus | C-O sole | Pleuronichthys coenosus |  | both | y |
| Enophrys bison | Buffalo Sculpin | Enophrys bison |  | both | y |
| Rimicola muscarum | Kelp clingfish | Rimicola muscarum |  | both | y |
| Trichodon trichodon | Pacific sandfish | Trichodon trichodon |  | both | y |
| Lepidopsetta1 | Rock and northern rock sole | Lepidopsetta bilineata, Lepidopsetta polyxystra | eDNA | both | y |
| Hypomesus pretiosus | Surf smelt | Hypomesus pretiosus |  | seine | y |
| Hexagrammos stelleri | Whitespotted greenling | Hexagrammos stelleri |  | seine | y |
| Chitonotus pugetensis | Striped kelpfish | Chitonotus pugetensis |  | seine | y |
| Rhinogobiops nicholsii | Blackeye goby | Rhinogobiops nicholsii |  | seine | y |
| Gadus macrocephalus | Pacific cod | Gadus macrocephalus |  | seine | y |
| Pallasina barbata | Tubenose poacher | Pallasina barbata |  | seine | y |
| Odontopyxis trispinosa | Pygmy poacher | Odontopyxis trispinosa |  | seine | y |
| Lepidogobius lepidus | Bay goby | Lepidogobius lepidus |  | seine | y |
| Oligocottus rimensis | Saddleback sculpin | Oligocottus rimensis |  | seine | n |
| Clinocottus acuticeps | Sharpnose sculpin | Clinocottus acuticeps |  | seine | y |
| Gibbonsia metzi | Striped kelpfish | Gibbonsia metzi |  | seine | y |
| Sebastes paucispinis | Bocaccio rockfish | Sebastes paucispinis |  | seine | y |
| Oncorhynchus mykiss | Rainbow trout | Oncorhynchus mykiss |  | eDNA | n |
| Merluccius productus | North Pacific hake | Merluccius productus |  | eDNA | n |
| Oncorhynchus clarkii | Cutthroat trout | Oncorhynchus clarkii |  | eDNA | n |
| Liparis mucosus | Slimy snailfish | Liparis mucosus |  | eDNA | n |
| Phytichthys chirus | Ribbon prickleback | Phytichthys chirus |  | eDNA | n |
| Mola mola | Ocean sunfish | Mola mola |  | eDNA | n |
| Clevelandia ios | Arrow goby | Clevelandia ios |  | eDNA | y |
| Hydrolagus colliei | Spotted ratfish | Hydrolagus colliei |  | eDNA | y |
| Icosteus aenigmaticus | Ragfish | Icosteus aenigmaticus |  | eDNA | n |
| Nautichthys oculofasciatus | Sailfin Sculpin | Nautichthys oculofasciatus |  | eDNA | n |
| Microstomus pacificus | Pacific dover sole | Microstomus pacificus |  | eDNA | n |
| Liparis florae | Tidepool snailfish | Liparis florae |  | eDNA | n |
| Clinocottus globiceps | Mosshead sculpin | Clinocottus globiceps |  | eDNA | n |
| Gibbonsia montereyensis | Crevice kelpfish | Gibbonsia montereyensis |  | eDNA | n |
| Anarrhichthys ocellatus | Wolf eel | Anarrhichthys ocellatus |  | eDNA | n |
| Engraulis mordax | Northern anchovy | Engraulis mordax |  | eDNA | n |
| Sebastes nebulosus | China rockfish | Sebastes nebulosus |  | eDNA | n |
| Sebastes entomelas | Widow rockfish | Sebastes entomelas |  | eDNA | n |
| Cottus1 | Coastrange and slimy sculpin | Cottus aleuticus, Cottus cognatus | eDNA | eDNA | n |
| Liparis cyclopus | Ribbon snailfish | Liparis cyclopus |  | eDNA | n |
| Polypera greeni | Lobefin snailfish | Polypera greeni |  | eDNA | n |
| Oncorhynchus keta | Chum salmon | Oncorhynchus keta |  | eDNA | n |
| Hippoglossus stenolepis | Pacific halibut | Hippoglossus stenolepis |  | eDNA | n |
| Xiphister1 | Black and rock prickleback | Xiphister atropurpureus, Xiphister mucosus | eDNA | eDNA | n |
| Cottus asper | Prickly sculpin | Cottus asper |  | eDNA | n |
| Oncorhynchus gorbuscha | Pink salmon | Oncorhynchus gorbuscha |  | eDNA | n |
