# Supplementary material for: Effect of environmental DNA sampling resolution in detecting nearshore fish biodiversity compared to capture surveys
Source: PeerJ. 2024 Oct 14;12:e17967. doi: 10.7717/peerj.17967 (PMC11485132; doi:10.7717/peerj.17967)
Supplement: Supplemental Information 13 — Variable descriptions found in Supplementary S4. * Indicates variables that were measured as overwater distance to that habitat feature. 1000-m radius and 100-m radius are the number of habitats (e.g., richness of seagrass, kelp, rocky shore, deep water, and freshwater habitats) found within 1000-m and 100-m radii. [file peerj-12-17967-s013.docx]

| ID | Hypothesis | Variables |
| --- | --- | --- |
| 1 | Proximity to vegetation habitats | seagrass* + kelp* |
| 2 | proximity to bathymetric habitats | rocky shore* + deep water (>25m)* + subtidal slope |
| 3 | proximity to freshwater | freshwater* |
| 4 | Habitat richness | 1000-m radius + 100-m radius |
| 5 | Seawater turnover | silt percent |
| 6 | Temporal offset | days between paired sampling |
| 7 | Habitat richness and seawater turnover | 1000-m radius + 100-m radius + silt percent |
