# Supplementary material for: Effect of environmental DNA sampling resolution in detecting nearshore fish biodiversity compared to capture surveys
Source: PeerJ. 2024 Oct 14;12:e17967. doi: 10.7717/peerj.17967 (PMC11485132; doi:10.7717/peerj.17967)
Supplement: Supplemental Information 14 — These variables were used to model both differences in richness and the ratio between turnover and nestedness. [file peerj-12-17967-s014.docx]

| **Group** | **Variable** | **Details** | **Code** |
| --- | --- | --- | --- |
| Habitat proximity | distance to canopy forming kelp | overwater distance to feature (meters) | kelp |
|  | distance to seagrass bed | overwater distance to feature (meters) | seagrass |
|  | distance to water >25-m deep | overwater distance to feature (meters) | water25m |
|  | distance to freshwater source | overwater distance to feature (meters) | freshwater |
|  | distance to rockyshore | overwater distance to feature (meters) | rockyshore |
|  | subtidal slope | slope below low tide (degrees) | slope_intertidal |
| Habitat richness | habitat features within 100 meters | number of features (above) within 100 meters | h100m |
|  | habitat features within 1000 meters | number of features (above) within 1000 meters | h1000m |
| Seawater turnover | percent fine sediment | % < 64 um | silt_percent |
| Temporal offset | days between paired sampling | Absolute number of days between eDNA and beach seine surveys of a site | dat_diff |
