# Supplementary material for: Effect of environmental DNA sampling resolution in detecting nearshore fish biodiversity compared to capture surveys
Source: PeerJ. 2024 Oct 14;12:e17967. doi: 10.7717/peerj.17967 (PMC11485132; doi:10.7717/peerj.17967)
Supplement: Supplemental Information 15 — Abbreviations: seagrass (distance to nearest seagrass bed), kelp (distance to nearest canopy forming kelp bed), rockyshore (distance to nearest cobble/boulder/bedrock shoreline), water25m (distance to water 25 meters deep), slope_subtidal (subtidal slope of sea floor), freshwater (distance to nearest freshwater output), h1000m (number of habitat features within 1000-m radius), h100m (number of habitat features within 100-m radius), and silt_percent (percent sediment > 64 µm). [file peerj-12-17967-s015.docx]

| **Hypothesis** | **Variables** | **df** | **logLik** | **AICc** | **delta** | **AICw** |
| --- | --- | --- | --- | --- | --- | --- |
| Habitat proximity | seagrass + kelp | 10 | -151.3 | 328.8 | 38.13 | 0.00 |
|  | rockyshore + water25m + slope_subtidal | 11 | -141.5 | 312.7 | 22 | 0.00 |
|  | freshwater | 9 | -148.0 | 319.0 | 28.3 | 0.00 |
| Habitat diversity | h1000m + h100m | 10 | -136.8 | 299.9 | 9.235 | 0.01 |
| Seawater turnover | silt_percent | 9 | -135.9 | 294.9 | 4.162 | 0.11 |
| Temporal offset | Dat_diff | 9 | -142.28 | 307.5 | 16.860 | 0.00 |
| Habitat diversity and seawater turnover | h100m + h1000m + silt_percent | 11 | -130.5 | 290.7 | 0 | 0.88 |
