# Supplementary material for: Effect of environmental DNA sampling resolution in detecting nearshore fish biodiversity compared to capture surveys
Source: PeerJ. 2024 Oct 14;12:e17967. doi: 10.7717/peerj.17967 (PMC11485132; doi:10.7717/peerj.17967)
Supplement: Supplemental Information 16 [file peerj-12-17967-s016.docx]

| Covariates | Unstandardized Coefficients | | Standardized Coefficients | t-value | p-value |
| --- | --- | --- | --- | --- | --- |
|  | B | Std. Error | Beta |  |  |
| Intercept | 5.501 | 5.625 | 10.066 | 0.978 | 0.334 |
| # of features within 100m | -2.125 | 0.857 | -2.720 | -2.480 | 0.017 |
| # of features within 1000m | 2.594 | 1.433 | 1.912 | 1.801 | 0.078 |
| % fine sediment | -47.371 | 12.769 | -4.284 | -3.710 | 0.001 |
| R^2 = 0.277, RMSE = 5.574, Sigma = 6.131 | | | | | |
