# Supplementary material for: Effect of environmental DNA sampling resolution in detecting nearshore fish biodiversity compared to capture surveys
Source: PeerJ. 2024 Oct 14;12:e17967. doi: 10.7717/peerj.17967 (PMC11485132; doi:10.7717/peerj.17967)
Supplement: Supplemental Information 17 — Variables were (pairwise distance-over-water), physical ocean exposure class, and vegetation percent cover. Abbreviations: distance (pairwise distance-over-water), exposure (physical ocean exposure class), veg. cover (vegetation percent cover), and date (Julian date starting January 1, 2018). [file peerj-12-17967-s017.docx]

|  |  | whole model | | | | | | | | single var. model |
| --- | --- | --- | --- | --- | --- | --- | --- | --- | --- | --- |
| Method | Variable | % deviance lost | p-value | % deviance explained | null deviance | GDM deviance | intercept | RMSE | observed - predicted correlation | % deviance explained |
| eDNA | distance | 32.02 | 0.000 | 25.3 | 91.1 | 68.1 | 0.26 | 0.13 | 0.47 | 20.0 |
|  | exposure | 15.98 | 0.004 |  |  |  |  |  |  | 14.9 |
|  | veg. cover | 1.00 | 0.540 |  |  |  |  |  |  | 1.4 |
|  | date | 3.86 | 0.260 |  |  |  |  |  |  | 0.6 |
| Beach seine | distance | 0.84 | 0.000 | 17.4 | 125.0 | 103.0 | 0.58 | 0.18 | 0.45 | 1.1 |
|  | exposure | 18.02 | 0.106 |  |  |  |  |  |  | 4.4 |
|  | veg. cover | 64.90 | 0.010 |  |  |  |  |  |  | 13.1 |
|  | date | 2.35 | 0.770 |  |  |  |  |  |  | 0.1 |
