# Supplementary material for: Effect of environmental DNA sampling resolution in detecting nearshore fish biodiversity compared to capture surveys
Source: PeerJ. 2024 Oct 14;12:e17967. doi: 10.7717/peerj.17967 (PMC11485132; doi:10.7717/peerj.17967)
Supplement: Supplemental Information 18 — Abbreviations: distance (pairwise distance-over-water), exposure (physical ocean exposure class), veg. cover (vegetation percent cover), and date (Julian date starting January 1, 2018). [file peerj-12-17967-s018.docx]

|  |  | whole model | | | | | | | | single var. model |
| --- | --- | --- | --- | --- | --- | --- | --- | --- | --- | --- |
| Method | Variable | % deviance lost | p-value | % deviance explained | null deviance | GDM deviance | intercept | RMSE | observed - predicted correlation | % deviance explained |
| eDNA | distance | 28.58 | 0.000 | 23.55 | 110.00 | 84.20 | 0.19 | 0.15 | 0.46 | - |
|  | exposure | 21.98 | 0.016 |  |  |  |  |  |  | - |
|  | veg. cover | 0.45 | 0.670 |  |  |  |  |  |  | - |
|  | date | 2.94 | 0.458 |  |  |  |  |  |  | - |
| Beach seine | distance | 5.73 | 0.000 | 16.78 | 138.00 | 115.00 | 0.04 | 0.18 | 0.38 | - |
|  | exposure | 14.41 | 0.048 |  |  |  |  |  |  | - |
|  | veg. cover | 51.96 | 0.006 |  |  |  |  |  |  | - |
|  | date | 2.38 | 0.570 |  |  |  |  |  |  | - |
