# Supplementary material for: Effect of environmental DNA sampling resolution in detecting nearshore fish biodiversity compared to capture surveys
Source: PeerJ. 2024 Oct 14;12:e17967. doi: 10.7717/peerj.17967 (PMC11485132; doi:10.7717/peerj.17967)
Supplement: Supplemental Information 19 — For each taxa the method of detection is provided along with the expected use (y/n) of sampled habitat (nearshore soft sediment). [file peerj-12-17967-s019.docx]

| lowest identifiable taxa | method of detection | expected |
| --- | --- | --- |
| Anoplopoma fimbria | eDNA | y |
| Brosmophycis marginata | eDNA | n |
| Cottus perplexus | eDNA | n |
| Cryptacanthodes giganteus | eDNA | y |
| Eopsetta jordani | eDNA | y |
| Glyptocephalus zachirus | eDNA | y |
| Hemilepidotus spinosus | eDNA | n |
| Hippoglossoides elassodon | eDNA | y |
| Icelinus borealis | eDNA | y |
| Icichthys lockingtoni | eDNA | n |
| Liparis fucensis | eDNA | y |
| Liparis pulchellus | eDNA | y |
| Lyopsetta exilis | eDNA | y |
| Oncorhynchus nerka | eDNA | n |
| Orthonopias triacis | eDNA | n |
| Prosopium williamsoni | eDNA | n |
| Rhamphocottus richardsonii | eDNA | n |
| Ronquilus jordani | eDNA | n |
| Scomber japonicus | eDNA | n |
| Sebastes mystinus | eDNA | n |
| Sebastes nigrocinctus | eDNA | y |
| Chitonotus pugetensis | beach seine and eDNA | y |
| Clinocottus acuticeps | beach seine and eDNA | y |
| Gadus macrocephalus | beach seine and eDNA | y |
| Hypomesus pretiosus | beach seine and eDNA | y |
| Lepidogobius lepidus | beach seine and eDNA | y |
| Odontopyxis trispinosa | beach seine and eDNA | y |
