# Supplementary material for: Effect of environmental DNA sampling resolution in detecting nearshore fish biodiversity compared to capture surveys
Source: PeerJ. 2024 Oct 14;12:e17967. doi: 10.7717/peerj.17967 (PMC11485132; doi:10.7717/peerj.17967)
Supplement: Supplemental Information 20 [file peerj-12-17967-s020.docx]

| Covariates | Unstandardized Coefficients | | Standardized Coefficients | t-value | p-value |
| --- | --- | --- | --- | --- | --- |
|  | B | Std. Error | Beta |  |  |
| Intercept | 20.915 | 6.567 | 10.066 | 3.185 | 0.003 |
| # of features within 100m | -1.568 | 0.996 | -2.720 | -1.574 | 0.123 |
| # of features within 1000m | 1.980 | 1.665 | 1.912 | 1.190 | 0.241 |
| % fine sediment | -55.939 | 15.363 | -4.284 | -3.641 | 0.000 |
| R^2 = 0.168, RMSE = 7.329, Sigma = 7.731 | | | | | |
