# Supplementary material for: Effect of environmental DNA sampling resolution in detecting nearshore fish biodiversity compared to capture surveys
Source: PeerJ. 2024 Oct 14;12:e17967. doi: 10.7717/peerj.17967 (PMC11485132; doi:10.7717/peerj.17967)
Supplement: Supplemental Information 21 [file peerj-12-17967-s021.docx]

|  | beach seine | | | eDNA (occupancy model) | | | eDNA (no occupancy model) | | |
| --- | --- | --- | --- | --- | --- | --- | --- | --- | --- |
| feature | S | rho | p-value | S | rho | p-value | S | rho | p-value |
| # of features within 100m | 483.5 | 0.50 | 0.030 | 1041.5 | -0.07 | 0.760 | 927.7 | 0.04 | 0.870 |
| # of features within 1000m | 1262.2 | -0.30 | 0.222 | 1072.4 | -0.11 | 0.670 | 1123.6 | -0.16 | 0.530 |
| % fine sediment | 867.8 | 0.10 | 0.680 | 1491.2 | -0.54 | 0.020 | 1582.6 | -0.63 | 0.004 |
