# Supplementary material for: Effect of environmental DNA sampling resolution in detecting nearshore fish biodiversity compared to capture surveys
Source: PeerJ. 2024 Oct 14;12:e17967. doi: 10.7717/peerj.17967 (PMC11485132; doi:10.7717/peerj.17967)
Supplement: Supplemental Information 22 — Variables were (pairwise distance-over-water), physical ocean exposure class, and vegetation percent cover. [file peerj-12-17967-s022.docx]

|  |  | whole model | | | | | | | | single var. model |
| --- | --- | --- | --- | --- | --- | --- | --- | --- | --- | --- |
| Method | Variable | % deviance lost | p-value | % deviance explained | null deviance | GDM deviance | intercept | RMSE | observed - predicted correlation | % deviance explained |
| eDNA | distance | 12.19 | 0.000 | 10.8 | 70.6 | 63.0 | 0.03 | 0.11 | 0.30 | 6.21 |
|  | exposure | 34.60 | 0.004 |  |  |  |  |  |  | 7.45 |
|  | veg. cover | 10.10 | 0.236 |  |  |  |  |  |  | 1.67 |
|  | date | 1.22 | 0.608 |  |  |  |  |  |  | 0.19 |
| Beach seine | distance | 0.69 | 0.000 | 18.1 | 131.0 | 107.0 | 0.58 | 0.18 | 0.39 | 1.06 |
|  | exposure | 18.35 | 0.088 |  |  |  |  |  |  | 4.32 |
|  | veg. cover | 66.30 | 0.008 |  |  |  |  |  |  | 13.70 |
|  | date | 1.80 | 0.700 |  |  |  |  |  |  | 0.14 |
