# Supplementary material for: Effect of environmental DNA sampling resolution in detecting nearshore fish biodiversity compared to capture surveys
Source: PeerJ. 2024 Oct 14;12:e17967. doi: 10.7717/peerj.17967 (PMC11485132; doi:10.7717/peerj.17967)
Supplement: Supplemental Information 23 — Details regarding (1) Biomass and Biomass density estimates from beach seining, (2) sample processing and PCR amplification, (3) Bioinformatics, quality control and taxonomic assignment, and (4) Select results, figures, and statistics of analyses without occupancy modeling. [file peerj-12-17967-s023.docx]

**Supplemental Text 1: Biomass and Biomass density estimates from beach seining.**

We estimated the biomass of each fish (n = 3364) that was measured using length-weight relationships retrieved from fishbase (Froese, Thorson & Reyes Jr, 2014). Total biomass per species at each site was estimated as the sum of individual weights of all measured individuals. For cases of highly abundant species (>20 individuals), subsamples were measured for length (and weight estimated as above), the weight distribution of the measured individuals was assumed to be representative of the whole sample, and the mean weight used to estimate total biomass (n = 1200). For cases where fish were not measured at all at a site (n = 133), we assumed the biomass distribution of unsampled individuals to match that of sampled individuals of conspecifics and averaged subsamples from survey-wide biomass distributions of each species. To account for differences in the area sampled among sites and sampling periods (mean = 605 m2, SD = 337 m2), biomass density (grams/ m2) was estimated by dividing the total estimated biomass of each species at site by the area sampled by the net.

**Supplemental Text 2: Details of sample processing and PCR amplification.**

All genetic sample processing was carried out in separate clean rooms specifically designed for extracting and amplifying eDNA samples. We extracted genetic material from the filters using a phenol-chloroform extraction procedure (Hawley et al., 2017). Library preparation followed (Miya et al., 2015), using a two-step PCR approach. In the first PCR, we used 12.5 µl of 2x Taq Froggamix (Froggabio), 0.6 µl of each primer at 10 µM (MiFish-U-F and MiFish-U-R), 2 µl of eDNA, and topped up the final volume to 25 µl using nuclease-free water. All the reactions were prepared in triplicates, and ran under the same thermal cycler conditions - initial denaturation at 95°C for 3 min, a three-step temperature repeated for 40 cycles (95°C for 30s, 63°C for 30s, 72°C for 30s), and a final extension at 72°C for 7 min. All three replicates were pooled and cleaned using SPRI beads (Beckman Coulter) at 0.8x volume. For the second PCR, also called indexing PCR, we used 12.5 µl of 2x Taq Froggamix (Froggabio), 2.5 µl of combinatory indexes (Nextera XT V2, sets A, B, C and D), 2.5 µl of DNA template (first PCR), and topped up the reaction to 25 µl for a final volume. The cycling conditions consisted of an initial denaturation at 95°C for 3 min, followed by 8 cycles with 3 temperatures for 30 seconds each (95°C, 55°C and 72°C), and a final extension at 72°C for 7 minutes. The indexed samples were again cleaned using the SPRI beads and quantified using the Quant-IT dsDNA HS kit (Invitrogen). We pooled the samples at equimolar concentration. As the final pool had two size fragments, one containing the 12SrRNA gene targeting fish species, and another one containing bacterial DNA, we performed an additional purification using a gel excision protocol. We purified the gel using the Wizard SV gel and purification clean-up system (Promega) and sequenced the resulting library on a MiSeq instrument (Illumina) in three separate runs using a 600 cycles V3 Reagent Kit.

**Supplemental Text 3: Bioinformatics, quality control and taxonomic assignment.**

We processed paired-end metabarcoding reads using the dada2 library v1.22.0 (Callahan et al., 2016) in R v4.1.3 (R Core Team, 2023), and used cutadapt v3.7 (Martin, 2011) to trim primers. We filtered primer-trimmed reads using default dada2 parameters, except reads were truncated to 180bp for forward and 150bp for reverse reads (truncLen) and maxEE was set to 4 for forward reads and 6 for reverse reads. We removed ASVs that had both a relative abundance of <= 0.1% and were detected only in a single sample.

After primer removal, quality trimming, amplicon sequence variant (ASV) inference, read merging, chimera removal, and noise reduction, we retained 24,629,880 reads in 955 ASVs. As an additional step to check for contamination, we calculated Bray-Curtis dissimilarity amongst field replicates from the same sampling event to identify and remove sites with > 0.8 dissimilarity ((O’Donnell et al., 2017); none were identified). We removed one site survey that had very low read depth (<5000) and rarified the remaining to a depth of 23,988 reads (the lowest read depth among remaining samples) to allow comparison between samples, resulting in 3,310,344 reads across 953 ASVs. In this step one ASV was removed entirely and 53 observations of ASVs (non-zero read counts at sites) were reduced to zeros. To estimate the probability of occurrence of each site-level ASV observation, we fit occupancy models (Royle & Link, 2006) to sample replicates within and across sites for each species. We then removed observations where the probability of occurrence at a site was <80% (Kelly et al., 2017), resulting in 3,078,676 reads across 107 ASVs. Of the 842 ASVs removed this way 737 were singleton observations (ASVs only detected once in the entire survey) and 105 were non-fish taxa. We have now clarified this in the Supplemental text.

We queried ASVs against the NCBI nucleotide database, based on identity percentage and e-values (number of times there were multiple species with equally highest values). We retrieved the top 10 taxonomic identities with greater than 96% match. Of these 107 ASVs, 8 were assigned to non-target taxa (Bacteria, Bacillariophyta, Balaenidae, and Bovidae; read depth: 3202) and were removed. The remaining 99 were assigned to fish (Actinopterygii and Chondrichthyes, read depth: 3,075,474).

To assign taxonomy to each ASV, we identified all equally top-hitting BLAST results that are fish known to exist in the Northeast Pacific Ocean (Alaska to southern California). For this we identified and excluded top-hitting species that have not been observed within 2000 km of our study area, using occurrence data in Aquamaps (Kaschner, K., Kesner-Reyes, K., Garilao, C., Segschneider, J., Rius-Barile, J. Rees, T., & Froese, R., 2019) – after which all top hitting results for each ASV contained at least one regionally expected species. When an ASV was assigned to multiple within-region species with an equal percent identity, we grouped those species together throughout the dataset as a partial identification in a manner analogous to partial identifications in the beach seining surveys. To assess within-taxon read number across sites and compare with biomass estimates, we calculated an eDNA read index as the read abundance within each sample relative to the maximum read abundance for each taxon (Port et al., 2016). This index has been shown to provide for a more robust estimate of relative abundance within a species compared to raw read abundance (Kelly, Shelton & Gallego, 2019).

**Supplemental Text 4: Select results, figures, and statistics of analyses without occupancy modeling.**

We present in this supplemental text select results, figures, and statistics, where the occupancy modelling step has been removed from the bioinformatics steps. This was done to weigh the effect that our data cleaning efforts had on our results. All other data processing and analyses remain the same as in the main text.

A similar pattern is found in false negatives is found in biomass density distributions (Figure S6), where errors are more prevalent when they were detected in low biomass densities with beach seining. There were fewer eDNA false-negatives (detected in beach seine only, Figure S7) and many more detections with eDNA only. These additional eDNA detections included 21 taxa novel to the study and known to inhabit the region as well as 6 taxa that were not detected in eDNA when using occupancy modeling (Table S9). The same top model of richness differences was found in the model selection procedure and similar effect sizes were found for each covariate (Table S10). However, the number of habitats within 100 m was not found to be significant (Table S10), where it had been when occupancy modeling was used. The effects of the number of habitats and percent silt on richness were similar (Figure S8, Table S11). Sub-regional clustering is not as apparent without occupancy modelling (Figure S10), however strong differences persist between exposed and protected sub-regions (PCoA Axis-1). This is further confirmed by the GDM results that show a weaker, yet significant effect of pairwise distance between sites, and a similar effect of physical exposure (Figure S10, Table S12).

This cursory comparison of methods suggests that in removing low-confidence observations via occupancy modeling some tentative true-positives are removed. However, occupancy modeling is used to remove tentative false-positives occurring from, among other things, potential environmental or cross-contamination. These low-confidence observations, when retained, may obscured patterns in beta diversity, such as this comparison suggests.

**Supplemental Text 5: Supplemental references.**

Batt MJ, Bennett-Steward DK, Couturier MC, Hammell DL, Harvey-Clark DC, Kreiberg MH, Iwama DG, Lall DS, Litvak DM, Rainnie DD, Stevens DD, Wright DJ, Griffin DG. CCAC guidelines on: the care and use of fish in research, teaching and testing, 2005.

Callahan BJ, Mcmurdie PJ, Rosen MJ, Han AW, Johnson AJA, Holmes SP. 2016. DADA2 : High-resolution sample inference from Illumina amplicon data. 13. DOI: 10.1038/nmeth.3869.

Froese R, Thorson JT, Reyes Jr RB. 2014. A Bayesian approach for estimating length-weight relationships in fishes. *Journal of Applied Ichthyology* 30:78–85. DOI: 10.1111/jai.12299.

Hawley AK, Torres-Beltrán M, Zaikova E, Walsh DA, Mueller A, Scofield M, Kheirandish S, Payne C, Pakhomova L, Bhatia M. 2017. A compendium of multi-omic sequence information from the Saanich Inlet water column. *Scientific data* 4:1–11.

Kaschner, K., Kesner-Reyes, K., Garilao, C., Segschneider, J., Rius-Barile, J. Rees, T., & Froese, R. 2019. AquaMaps: Predicted range maps for aquatic species.

Kelly RP, Closek CJ, O’Donnell JL, Kralj JE, Shelton AO, Samhouri JF. 2017. Genetic and manual survey methods yield different and complementary views of an ecosystem. *Frontiers in Marine Science* 3:1–11. DOI: 10.3389/FMARS.2016.00283.

Kelly RP, Shelton AO, Gallego R. 2019. Understanding PCR Processes to Draw Meaningful Conclusions from Environmental DNA Studies. *Scientific Reports* 9:1–14. DOI: 10.1038/s41598-019-48546-x.

Martin M. 2011. Cutadapt removes adapter sequences from high-throughput sequencing reads. *EMBnet. journal* 17:10–12.

Miya M, Sato Y, Fukunaga T, Sado T, Poulsen JY, Sato K, Minamoto T, Yamamoto S, Yamanaka H, Araki H, Kondoh M, Iwasaki W. 2015. MiFish, a set of universal PCR primers for metabarcoding environmental DNA from fishes: Detection of more than 230 subtropical marine species. *Royal Society Open Science* 2. DOI: 10.1098/rsos.150088.

O’Donnell JL, Kelly RP, Shelton AO, Samhouri JF, Lowell NC, Williams GD. 2017. Spatial distribution of environmental DNA in a nearshore marine habitat. *PeerJ* 2017. DOI: 10.7717/peerj.3044.

Port JA, O’Donnell JL, Romero-Maraccini OC, Leary PR, Litvin SY, Nickols KJ, Yamahara KM, Kelly RP. 2016. Assessing vertebrate biodiversity in a kelp forest ecosystem using environmental DNA. *Molecular Ecology* 25:527–541. DOI: 10.1111/mec.13481.

R Core Team. 2023. R: A Language and Environment for Statistical. Computing. R Foundation for Statistical Computing.

Royle JA, Link WA. 2006. Generalized site occupancy models allowing for false positive and false negative errors. *Ecology* 87:835–841. DOI: 10.1890/0012-9658(2006)87[835:GSOMAF]2.0.CO;2.
